# Novel Inducers of the Envelope Stress Response BaeSR in *Salmonella* Typhimurium: BaeR Is Critically Required for Tungstate Waste Disposal

**DOI:** 10.1371/journal.pone.0023713

**Published:** 2011-08-23

**Authors:** Corinne Appia-Ayme, Elaine Patrick, Matthew J. Sullivan, Mark J. Alston, Sarah J. Field, Manal AbuOun, Muna F. Anjum, Gary Rowley

**Affiliations:** 1 School of Biological Sciences, University of East Anglia, Norwich Research Park, Norwich, United Kingdom; 2 Animal Health and Veterinary Laboratories Agency (AHVLA) (Weybridge), Surrey, United Kingdom; 3 The Genome Analysis Centre, Norwich Research Park, Norwich, United Kingdom; University of Birmingham, United Kingdom

## Abstract

The RpoE and CpxR regulated envelope stress responses are extremely important for *Salmonella*Typhimurium to cause infection in a range of hosts. Until now the role for BaeSR in both the *Salmonella* Typhimurium response to stress and its contribution to infection have not been fully elucidated. Here we demonstrate stationary phase growth, iron and sodium tungstate as novel inducers of the BaeRregulon, with BaeR critically required for *Salmonella* resistance to sodium tungstate. We show that functional overlap between the resistance nodulation-cell division (RND) multidrug transporters, MdtA, AcrD and AcrB exists for the waste disposal of tungstate from the cell. We also point to a role for enterobactinsiderophores in the protection of enteric organisms from tungstate, akin to the scenario in nitrogen fixing bacteria. Surprisingly, BaeR is the first envelope stress response pathway investigated in *S*. Typhimurium that is not required for murine typhoid in either ity^S^ or ity^R^ mouse backgrounds. BaeR is therefore either required for survival in larger mammals such as pigs or calves, an avian host such as chickens, or survival out with the host altogether where *Salmonella* and related enterics must survive in soil and water.

## Introduction

As an intracellular pathogen of cold and warm blooded animals *Salmonella* has to survive a range of environmental stress conditions both within and out with a host. The ability to do this is in part governed by highly responsive transcriptional regulators, which respond to specific stimuli and switch on a cascade of signalling events to counter the given stress. Environmental stresses, which damage the outer membrane, or disrupt perisplasmic homeostasis of Gram negative bacteria leads to stimulation of the envelope stress response (ESR) [1]. The ESR consists of a least 5 partially overlapping systems, the alternative sigma factor σ^E^ (RpoE) [2], [3], [4], the two component regulators CpxAR[2], [3], [4], [5], [6], [7] and BaeSR[8], the phage shock proteins (PspABCDEF) [9], [10], [11], [12], [13], [14] and the RcsCDBphosphorelay system [15].

Of these systems the best characterised in response to envelope stress in terms of both regulation and regulon members are those governed by σ^E^and CpxRA. σ^E^ responds to misfolded outer membrane proteins (OMPs) within the periplasm[16], [17], whilst CpxRA responds to overproduction of outer membrane lipoproteins or P pili[18]. σ^E^and CpxRA are both extremely important for a range of pathogens to cause infection, for review see [1]. Intrinsic differences between the envelope stress response pathways of laboratory strains of *E. coli* and pathogens such as *Salmonella* have been documented. For example, RpoE, even though an alternative sigma factor, is essential for cell viability in *E. coli* [19] but is not required for cell viability of *Salmonella* and a range of other pathogens under non-inducing conditions [20], [21], [22].

Possibly the least characterised ESR is that controlled by BaeSR. BaeSR was first identified in *E. coli* in a screen for novel two component systems [23]. It was then further characterised as the third ESR pathway behind σ^E^ and CpxAR[8], through screening for factors which controlled expression of the periplasmic protein Spy (spheroplast protein Y). Transcriptomic analyses to determine the BaeSR regulon in *E. coli*, have reached different conclusions, probably due to differences in strain type and overexpression conditions used [24], [25]. From these studies the core BaeSR regulon of *E. coli* appears to consist of *spy*, *mdtA*, *andacrD*.The physiological role of Spy, although very well conserved across Gram negatives bacteria, and its co-regulation by CpxRA, currently remains enigmatic, although recently the crystal structure of Spy and protein chaperone activity has been elegantly elucidated [26]. MdtA, AcrB and AcrD are all members of the resistance nodulation-cell division (RND) family of multidrug transporters. Overexpression of BaeR in a *ΔacrB* backgroundin *E. coli*upregulates*mdtA* and *acrD*and results in resistance to ß-lactam antibiotics, novobiocin and deoxycholate[27], [28]. The histidine kinase sensor, BaeS is not essential for the effect of overexpressed BaeR [27] indicating a degree of self phosphorylation of the response regulator BaeRor phosphorylation by another histidine kinase.

It is strange however, that if the primary role of BaeR is to maintain drug efflux, which are required for resistance to a plethora of different noxious agents, that there are so few reported phenotypes associated with loss of BaeR, asides from a sensitivity to indole, copper, zinc and tannins [8], [29], [30]. This may point to functional overlap between the envelope stress pathways. This occurs at the regulon level with BaeR and CpxR both regulating Spy, AcrD and MdtA, and RpoE and Cpx both regulating DegP, but may also occur as cross talk between two component systems, with some evidence of BaeS being able to trans-phosphorylate non-cognate response regulators CheY and YfhA [31] .The alternative hypothesis is that the correct physiological and/or environmental inducing conditions of BaeR have not yet been identified.

In this study, we characterise the role of *S*. Typhimurium BaeSR in terms of response to stress and host environment. We show for the first time that iron and stationary phase growth are novel inducers of BaeR activity. We clearly show that while activity of BaeR is induced by a high concentration of indole, a *baeR* deletion mutant is not growth inhibited by indole addition as reported in *E.coli*. In a two-pronged approach of transcriptomics and genomic screening, we identify tungstate as an environmental stress where BaeR is essential for both regulation of drug efflux and cell survival and demonstrate the combined role of MdtA, and either AcrD or AcrB in heavy metal detoxification. We also demonstrate that of the *S*. Typhimurium envelope stress pathways analysed thus far, BaeR is the first that is not required to establish murine typhoid.

## Results

### BaeSRis not required for *Salmonella* to establish murine Typhoid

**Figure 1:**
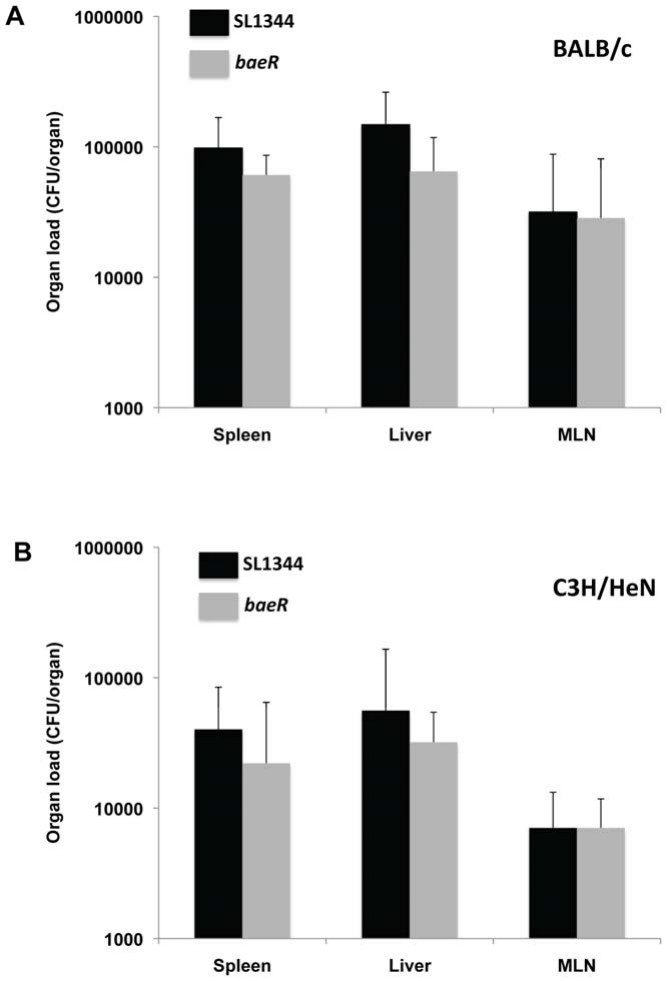
Oral infection of BALB/c and C3H/HeN mice. Mice were inoculated orally with either 5×10^5^ CFU of the WT strain or the*baeR*mutant and the number of bacteria present in different organs determined 5d later for BALB/c and 10d later for C3H/Hen mice. The bar represents the mean of 5 mice and the error bar indicates the SD.

ESR pathways are important for *S*. Typhimurium infection of mice and larger animals. Recently BaeSR has been shown to be required for *Salmonella* Dublin colonisation of orally infected calves[32]. To ascertain any involvement of BaeR in murine Typhoid, a competition assay was performed with an *S*. Typhimurium SL1344*baeR* mutant versus the isogenic parent strain (WT) in BALB/c mice. A dose containing equal numbers of the *baeR* and WT strains (2×10^3^ CFU/strain) was inoculated via the intraperitoneal route (IP) into mice, and CFU in livers and spleens enumerated 3 days later. The competitive index (CI) of almost 1 demonstrates that the *baeR* mutant is not significantly attenuated (data not shown). IP infection bypasses the normal infection route, and eliminates exposure to a number of conditions.As stress such as the acidic pH of the gut and an array of antimicrobial peptides secreted by the gut epithelia might induce envelope stress, oral infection of BALB/c mice was performed. Figure 1A depicts the organ load in the spleen, liver and mesenteric lymph nodes following oral infection with either the isogenic parent or *baeR* mutant, with no significant attenuation of the *baeR* mutant.

The phage shock pathway is only attenuated in ity^R^ mice, due to the importance of PspA in regulating transport of essential divalent metal cations required to combat the affects of the host natural resistance-associated macrophage protein 1 (Nramp1) [33]. BaeR has been reported to be involved in the response to the metals copper and zinc [29], [34]so we hypothesised that BaeR might only be required for infection of ity^R^mice akin to the situation with PspA. This scenario would also correlate with the role for BaeR in *Salmonella* Dublin colonisation of orally infected calves. However, the *baeR* mutant was not attenuated after oral infection of C3H/HeN mice (ity^R^) (Figure 1B). Consistent with these negative data, the *baeR* mutant is not detrimental to *Salmonella* Typhimurium invasion and intracellular replication of epithelial cells (HeLa) and macrophages (RAW264.7) respectively (data not shown).

### *Salmonella* BaeR is induced by indole, copper, iron and zinc but is not required for resistance

BaeSR was identified as the third envelope stress response pathway in *E. coli* [8], and shares overlap with CpxAR in regulation of the periplasmic protein Spy. We ascertained whether loss of BaeR in *S*. Typhimurium results in phenotypes characteristic of envelope damage in this organism. To determine this *baeS*, *baeR* and *baeSR* deletion mutants were exposed to a range of *in vitro* stress conditions, including high temperature (48^o^C), hydrogen peroxide and high salt. None of the *bae* mutants tested were any more sensitive to these conditions than the isogenic parent strain, SL1344 (data not shown).

**Figure 2:**
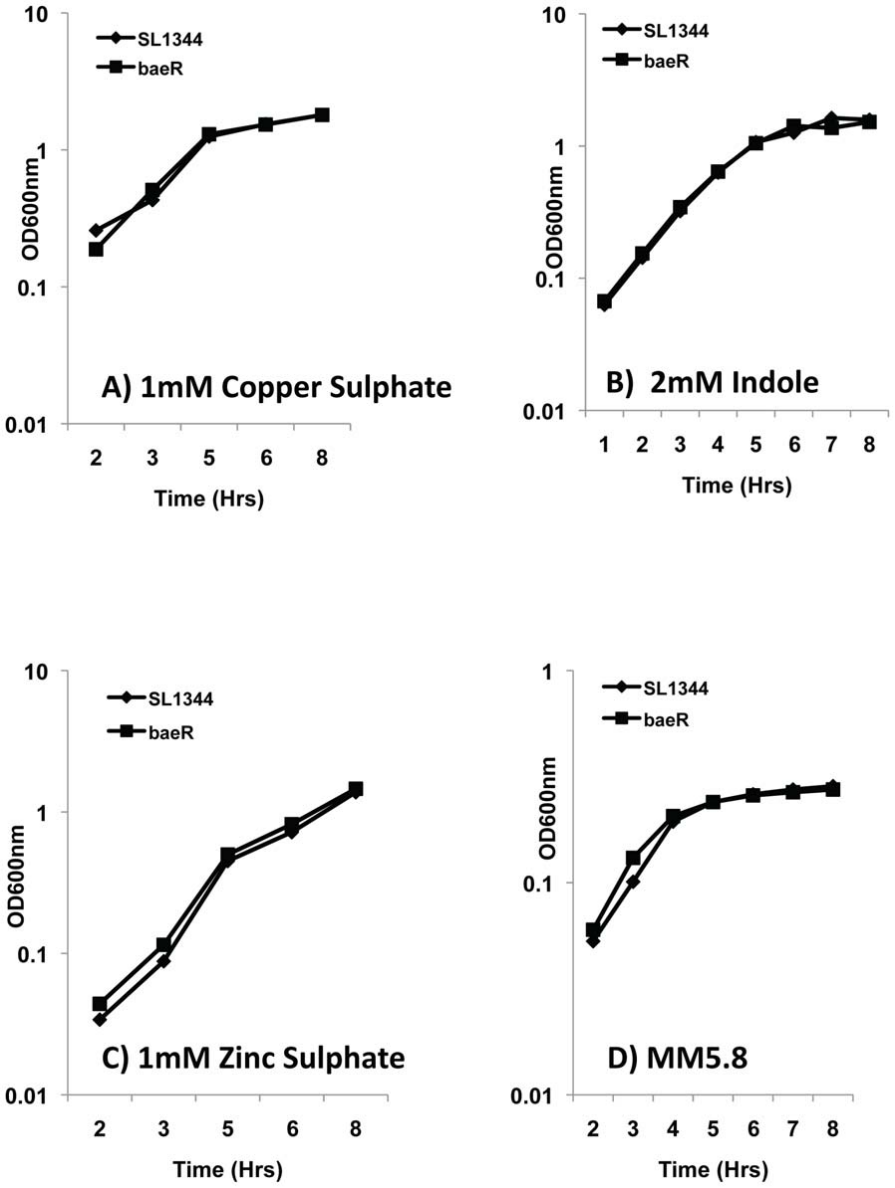
Growth curves of *Salmonella* Typhimurium SL1344 wild type and *baeR* mutant. LB media was supplemented with 1mM Copper Sulphate (A), 2mM Indole (B) and 1mM Zinc Sulphate (C). In panel (D), the cells were grown in Minimal Media pH5.8 to mimic intra-macrophages conditions. The growth of the *baeR* mutant was not significantly different from the wild type in either of those conditions.

**Figure 3:**
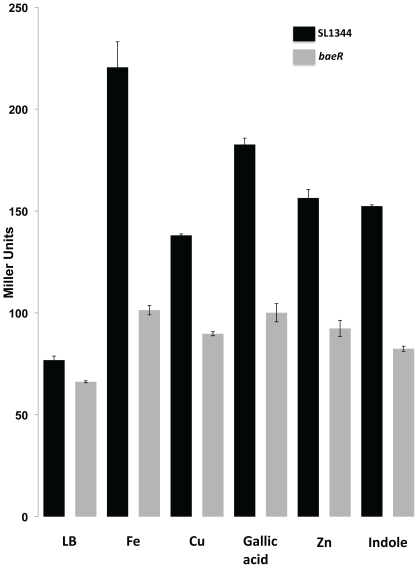
BaeR dependent induction of *mdtA*. β-galactosidase activities of the plasmid containing the *mdtA* promoter region fused with the promoter-less *lacZ* gene in a wild type (black columns) or *baeR* mutant (grey columns) background. The cells were grown in LB or LB supplemented with 1mM Iron Sulphate (Fe), 1mM Copper sulphate (Cu), 0.5mM Zinc Sulphate (Zn), 0.5mM of Gallic Acid or 2mM of Indole. Bars represent the mean of three biological replicates, error bars, SD.

Indole has been previously reported as an inducer of *baeR* expression in both *E. coli* and *Salmonella* [8], [29], with loss of *baeR* resulting in indole sensitivity in *E. coli* [8]. However, in our hands, a *S*. Typhimurium*baeR* mutant is not sensitive to 2mM indole. (Figure 2B). There is also conflicting evidence in the literature surrounding the induction of *baeR* dependent promoters by metals. [34]reported that out of 19 metals tested only zinc induced a *spy-lacZ* fusion in a BaeR-dependent fashion in *E. coli*. [29]supports the role of zinc but adds copper as an inducer of MdtA in a BaeR dependent manner in *S*. Typhimurium ATCC 14028and shows that a *baeSR* mutant in this same strain is growth inhibited by copper and zinc. No growth inhibition of our SL1344 *baeR* mutants in comparison to the isogenic parent could be detected over a range of concentrations of both zinc (0–4mM) and copper (0–6mM), with Figure 2 depicting this for a single concentration of each. Using an *mdtA-lacZ* promoter fusion, which contains a well characterised BaeR binding site [29], and in support of the literature, copper, zinc and indole do induce expression of *mdtA* in a BaeR dependent nature (Figure 3). However using *mdtA-lacZ* as a reporter of BaeR activity, we also tested a range of other metals similar to the studies in *E. coli* and determined that iron is a stronger inducer of *baeR* compared to both copper and zinc, with a 3 fold induction observed with iron, compared to 1.8 or 2 fold for copper and zinc respectively (Figure 3). The *baeR* mutant is not growth restricted by excess or depleted iron conditions (data not shown).

### BaeR is induced during stationary phase

**Figure 4:**
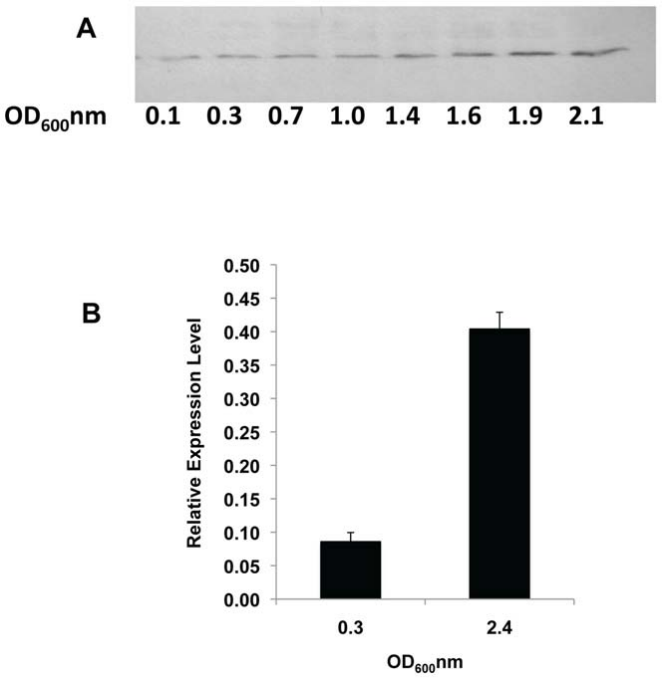
BaeR protein and mRNA are more abundant in stationary phase. Panel A: Immunoblot of BaeR-6His through growth showing that the protein is more abundant at the late stationary phase. Panel B: Quantitative RT-PCR showing the level of *baeR* mRNA in exponential phase (OD_600_ = 0.3) and late stationary phase (OD_600_ = 2.4). Level of expression is in arbitrary units and is normalised to *ampD* gene expression.

RpoE is induced during the stationary phase of LB cultures [35], and is also important for the starvation stress response [36]. To test whether BaeR is linked to a particular growth phase, western blot analysis of chromosomally epitope tagged BaeR was performed through growth of an aerated LB culture (Figure 4A). The amount of protein loaded on the SDS PAGE gels was normalised to OD_600_ and clearly shows an induction of BaeR upon entry into and during stationary phases of growth. To validate this induction of BaeR in stationary phase we compared transcript levels of *baeR* from mid-exponential and stationary phase cultures (Figure 4B). In agreement with the protein analysis *baeR* is clearly induced during stationary phase survival and indicates a role for BaeR under these conditions.

### A*S.* Typhimurium*baeR*mutantis sensitive to tungstate

To identify conditions where there is an absolute requirement for BaeR in *S*. Typhimurium physiology and response to stress, phenotype microarrayswere employed. Phenotype microarrays (PM) allows screening for almost 2000 phenotypes simultaneously, by measuring bacterial respiration in the presence of a variety of different nutrients or inhibitors [37]. A previous phenotype microarray study which analysed mutants in all of the two component systems in *E. coli*, including BaeSR, identified myricetin, gallic acid, nickel chloride and sodium tungstate as agents to which a *baeSR* mutant was more sensitive [38], although none of the sensitivity phenotypes were verified by alternative methods.

Due to the intrinsic differences identified between the ESR of *E. coli* and *Salmonella,* combined with the *in vitro* differences we have described for BaeR as reported above, we performed phenotype arrays directly on the*S*. Typhimurium*baeR* mutant. Duplicate phenotype array analysis using all 20 PM plates was performed on both the isogenic parent strain and the *S*. Typhimurium*baeR* mutant. Surprisingly for a system attributed to maintenance of the bacterial envelope, analysis of 1920 conditions, including an array of toxic compounds and antimicrobials, identified sodium tungstate as the only condition which resulted in growth suppression of the *S*. Typhimurium*baeR* mutant and not the wild type strain.

**Figure 5:**
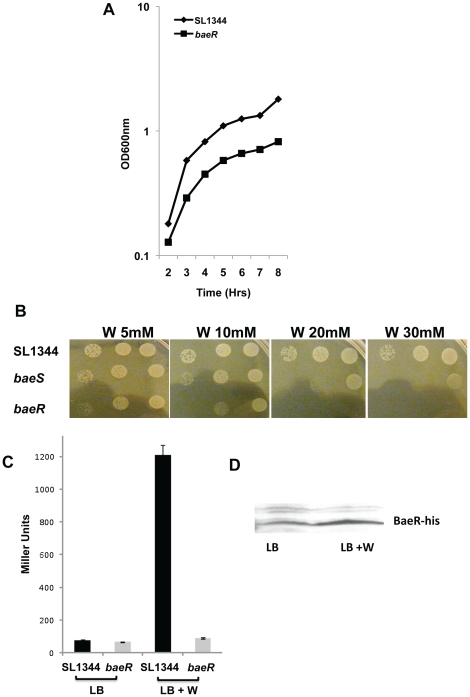
The growth of a *baeR* mutant is impaired on media containing tungstate and *mdtA* induction is BaeR dependent. Panel A: growth curve of SL1344 wild type and *baeR* mutant on LB complemented with 20mM of sodium tungstate. Panel B: SL1344 wild type, *baeS* and *baeR* mutant spotted on LB containing increasing concentrations on sodium tungstate (W). Panel C: β-galactosidase activities of the plasmid containing the *mdtA* promoter region fused with the promoter-less *lacZ* gene in a wild type (black columns) or *baeR* mutant (grey columns) background on LB or LB complemented with 20mM of sodium tungstate. Panel D: Immunoblot of BaeR-6His of *Salmonella* grown in LB with or without sodium tungstate.

**Figure 6:**
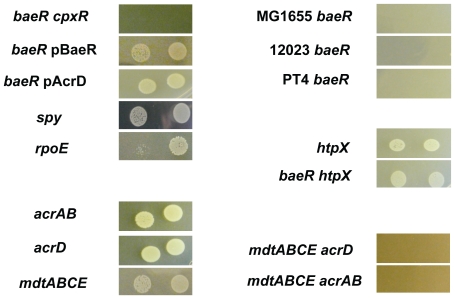
Tungstate sensitivity of *baeR* mutants in *E. coli* and *Salmonella* and of different *Salmonella* Typhimurium SL1344 mutants. Two concentrations of cells were spotted on LB agar plates containing 30mM of sodium tungstate and grown at 37°C overnight. MG1655 is an *E. coli* strain, 12023 is a *Salmonella* Typhimurium strain and PT4 is a *Salmonella* Enteritidis strain (see Table S1).

To confirm the observed tungstate sensitivity from the PM plates, the *baeR* mutant was exposed to tungstate both in liquid and solid phase growth (Figure 5A and B). In the presence of low concentrations of tungstate there are only subtle differences between the WT and the *baeR* mutant (figure 5B). However, significant differences in growth are clearly visible on plates containing 10mM tungstate, while on plates containing 20mM tungstate the *baeR* mutant is not viable. The isogenic parent strain is not growth inhibited even on plates even in the presence of 30mM tungstate. In liquid culture the *baeR*mutant is growth restricted at 20mM tungstate, but is still viable and is the concentration used for transcriptomic analysis below. Similar differences between survival of WT and the *baeR*mutant can be observed during anaerobic culture, but the concentration of tungstate required in plates is much higher, 60mM (data not shown). Complementation of *baeRin trans* fully restores growth on tungstate to that observed with the parent strain (Figure 6). The *baeS* mutant grows better than the *baeR* mutant in the presence of tungstate, indicating that there is sufficient auto-phosphorylation of BaeR, or cross talk with a non-cognate histidine senor kinase (Figure 5B). Loss of BaeR also results in tungstate sensitivity, in *Salmonella*Typhimurium12023,*Salmonella* Enteritidis PT4 and *E. coli* MG1655 backgrounds (Figure 6).

As well as sensitivity of the mutant to tungstate, *S*. Typhimurium*baeR* is clearly induced in the presence of tungstate when analysed by qRT-PCR and western blot (Figure 5C and D). Using the *mdtA-lacZ* fusion described earlier, as opposed to the 2 and 3 fold inductions observed with indole, zinc etc, in the presence of sodium tungstate there is a more than 10 fold induction of the *mdtA* reporter which is dependent on BaeR (figure 5C). This is the first environmental stress identified where BaeR is both induced and required for cell viability. We also tested a range of gallic acid concentrations in similar experiments. While we agree that gallic acid does induce *mdtA* in a BaeR-dependent manner (figure 3), BaeR is not required for *Salmonella* to survive in the presence of gallic acid under the conditions we have tested (data not shown).

### DNA microarray based identification of *Salmonella* tungstate responsive genes

**Figure 7:**
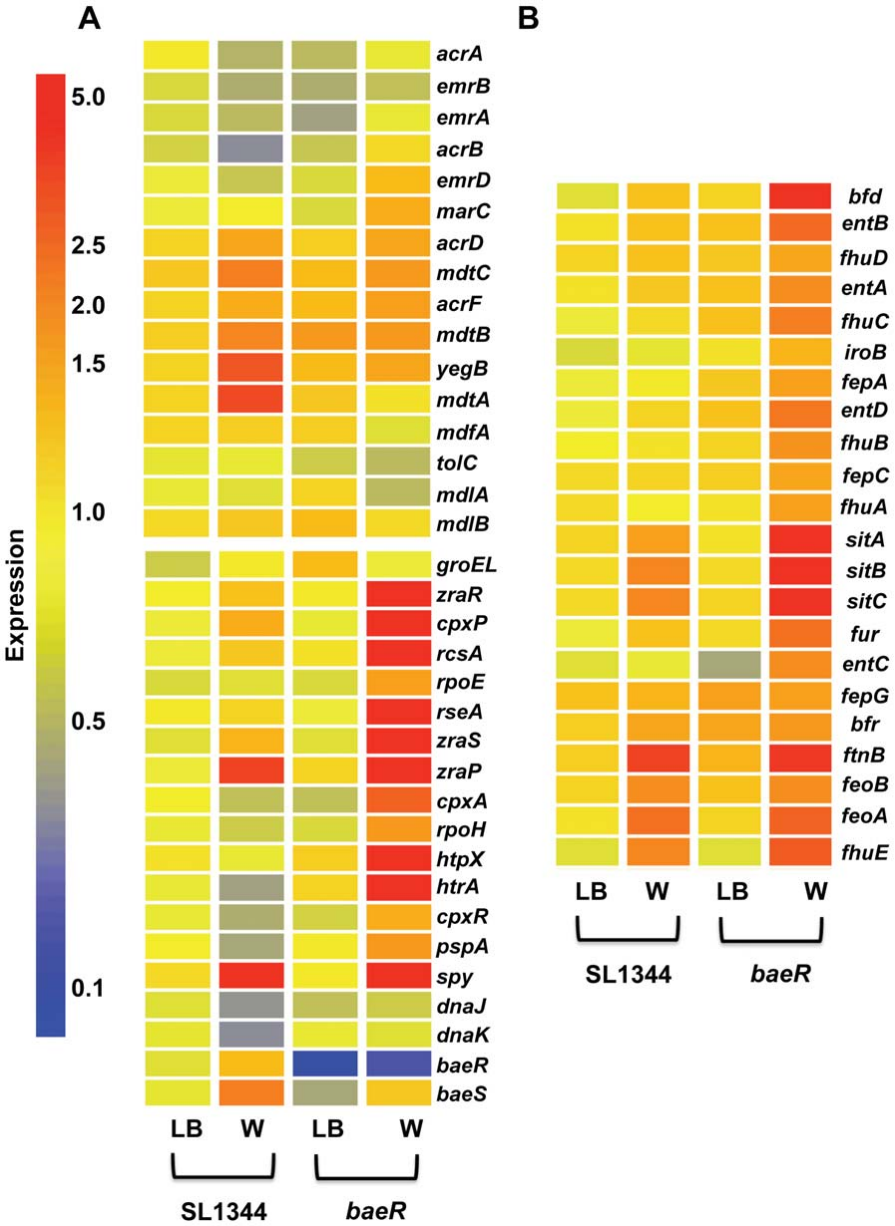
Heat map representing the expression level of selected genes of *Salmonella* wild type and *baeR* mutant in LB +/−20mM sodium tungstate. Panel A: genes involved in the transport and detoxification of metals and antibiotics (top panel) and genes involved in the response to stress, mainly envelope stress (bottom panel). Panel B: genes involved in the regulation and transport of iron and in the synthesis and transport of siderophores.

MdtA, AcrD, and Spy [8], [25], [27], [29] had already been identified as BaeR regulated in other studies so we first tested the sensitivity of mutants in these genes to tungstate. Figure 6 shows that each of these mutants is able to grow on 30mM Tungstate and individually do not account for the phenotype of the *baeR* mutant. As a tool to identify the *Salmonella* BaeR regulon and unpick the tungstate phenotype, DNA microarrays were employed to measure the global transcriptional differences between the WT and *baeR* mutant in the presence and absence of tungstate (DataS1). When the wild type strain of *Salmonella* is grown in the presence of 20mM tungstate, 87 genes are induced more than 2 fold (with 95% confidence using Rank Product statistical analysis) while 60 are repressed more than 2 fold compared with LB alone. Tungstate is a heavy metal that may interfere with the bacterial envelope and disturb the stability of metalloenzymes. We observe that some of the transcriptional changes are related to this proposed toxicity. For example, genes involved in drug efflux, envelope stress and DNA repair genes (Figure 7A and DataS1) were induced by the presence of tungstate. Likewise, the expression of genes encoding proteins involved in the folding and maturation of metal-containing enzymes such as the MoeABC and SufABCD systems were enhanced by the presence of tungstate (DataS1).

Of greater interest for this study are the genes that are transcriptionally dependent on BaeR. When cultured on LB, no gene is differentially expressed between the wild type and *baeR* mutant; if tungstate is added in the media, the BaeR regulon is significantly larger: 118 genes are up-regulated and 105 down regulated in a *baeR* background versus wild type *Salmonella*. The deletion of *baeR* seems to enhance the membrane stress related transcriptional response towards tungstate, *cpxAR*, *rcsA, rpoE*, *spy*, and *htpX*, encoding a stress controlled protease [39], are significanlty up regulated in a *baeR* mutant. Genes encoding a zinc responsive two-component regulator, *zraSR*, and an associated periplasmic protein, *zraP* are also significantly induced in a *baeR* mutant on tungstate (Figure 7A). In order to investigate the contribution of all other ESR system in the tungstate sensitivity, we tested the ability of ESR mutants to grown on tungstate (Table S1). Only an *rpoE* mutant has a slower growth on 30mM tungstate plates (Figure 6) but the extensive pleiotropic effects of an *rpoE* mutation could explain this phenotype. Surprisingly, growth of *baeR* on tungstate is restored in a *baeR htpX* double mutant (Figure 6), suggesting that the overproduction of HtpX could affect the fitness of the *baeR* mutant when sodium tungstate is present. A similar induction on tungstate can be observed for genes related to iron regulation, transport, siderophores synthesis and export such as the *ent* genes (Figure 7B). The phenotype of the *ent* mutants are discussed below. While drug efflux system such as *mdtA* and *acrD* were up-regulated in WT *Salmonella* plus tungstate, the level of up-regulation is not as great in the *baeR* mutant (figure 7A). However, as a possible compensatory mechanism for loss of *baeR* other efflux systems such as *mar* and *emr* are upregulated in a *baeR* mutant plus tungstate but not WT.

It has been difficult to relate the down regulation of some genes in a *baeR* mutant to its phenotype on tungstate. For instance, the *nap* and *nar* genes, encoding nitrate reductases, and the *nir* operon encoding a nitrite reductase are up-regulated in the presence of tungstate only if BaeR is present (Table 1 and DataS1). As deletion mutants in these genes can grow on tungstate (Table S1) and the encoded proteins are not necessary for growth in an aerobic rich media, their BaeR regulation in this condition is difficult to explain. The same conclusion is true for all the deletion mutants where expression is down regulated in the *baeR* mutant (Table S1). Their loss is not the cause of the *baeR* mutant phenotype in this growth condition, at least individually, but these genes may form part of the BaeR regulon.

**Table 1:** Real time quantitative PCR on selected target genes.

Target gene	WT in LB	*baeR*in LB	WT in LB+Tungstate	*baeR* in LB+Tungstate	Induction WT Tungstate/WT LB
*baeS*	0.12^+^/_−_0.03	0.26^+^/_−_0.01	0.30^+^/_−_0.05	0.23^+^/_−_0.00	2.26
*baeR*	0.10^+^/_−_0.03	*ND*	0.17^+^/_−_0.05	*ND*	1.70
*Spy*	0.79^+^/_−_0.03	0.76^+^/_−_0.36	42.13^+^/_−_11.5	81.69^+^/_−_25.32	53.32
*nirB*	0.15^+^/_−_0.03	0.19^+^/_−_0.01	4.58^+^/_−_0.05	0.04^+^/_−_0.00	30.53
*narG*	0.07^+^/_−_0.00	0.08^+^/_−_0.01	5.48^+^/_−_0.50	0.07^+^/_−_0.00	78.28
*napA*	0.34^+^/_−_0.03	0.52^+^/_−_0.03	2.64^+^/_−_0.14	0.05^+^/_−_0.00	7.76

Expression values are normalised to *ampD* gene expression for each RNA sample.

*ND*: not determined.

### RND type multidrug efflux pumps are required for tungstate waste disposal

To complement the microarray based approach, which would include both direct and indirect transcriptional changes caused by the loss of BaeR in the presence of tungstate, we also sought to randomly complement the tungstate *baeR* phenotype. A cosmid library containing large fragments of *E. coli* BL21 chromosome (20 kpb in average) was introduced in SL1344 *baeR* mutant by conjugation. More than 500 transformants were picked from the selection plates, grown over night and spotted on LB plates containing 30mM of sodium tungstate. The cosmids from the colonies growing on tungstate plates were purified and re-introduced in the *baeR* mutant. Within the six cosmids complementing the phenotype, four were harbouring the same DNA fragment, one harboured a fragment which overlapped with the four cosmids and one cosmid contained the *mdtABCDbaeSR* locus. Each gene present on the overlapping DNA fragment was cloned individually on a plasmid to test its ability to complement *baeR* mutant by itself. Only pAcrD was able to complement the growth of *baeR* on tungstate (Figure 6). Surprisingly, the growth on tungstate of an *acrD* mutant is not affected. Similarly, none of the genes encoding a multidrug efflux pump is essential for *Salmonella* growth on tungstate (Table S1). However, *Salmonella* strains mutated in both *mdtABCE* and*acrD,* or, *mdtABCE and acrAB*–but not *acrD* and *acrAB*- lose their capability to grow on LB agar supplemented with 30mM tungstate. In agreement with this overlapping function between the drug efflux pumps, a *S*. Typhimurium*tolC* mutant is also sensitive to tungstate. TolC alone cannot complement the *baeR* mutant, which rules out the possibility of disruption of the universal outer membrane barrel rather than the efflux structures themselves.

### Enterobactin siderophores protect *Salmonella* from Tungstate toxicity

**Figure 8:**
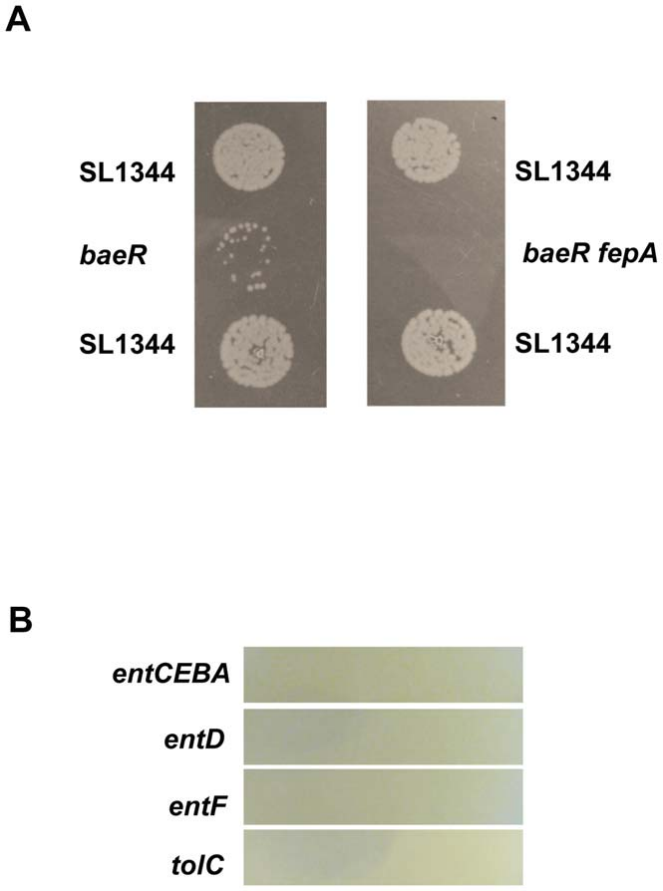
*baeR* growth on tungstate can be complemented by a secreted factor present in the wild type. Panel A: *baeR* isolated (bottom left of the photo) does not grow on sodium tungstate plates while the presence of the wild type strain in close proximity restore the growth (top of the photo), presumably by the secretion of siderophores rescuing the metal detoxification. A *baeRfepA* double mutant (right) cannot be rescued by the WT. Panel B: *Salmonella* deletion mutants in the genes involved in enterobactin synthesis and efflux cannot grow on LB agar plates with 30mM of sodium tungstate.

We have clearly demonstrated earlier in this study that BaeR is required for *Salmonella* survival in the presence of 30mM tungstate and have identified a BaeR dependent mechanism for the detoxification or waste disposal of tungstate, through regulation of RND type multidrug transporters. Waste disposal is a term that has already been attributed to the function of efflux pumps in *E. co*li and fits well with this scenario [40]. However when a *baeR* mutant is cultured on 30mM tungstate in between two wild type *Salmonella* cultures it grows as well as the parent (Figure 8A). This points to two possibilities; the wild type *Salmonella* alters the state of tungstate during the process of detoxification or that a secreted component also contributes to tungstate detoxification.

In N_2_-fixing bacteria such as *Azotobacter vinelandii* where tungstate is toxic when in large excess over molybdate, catechol siderophores, such as protochelin, control tungstate uptake and toxicity [41], [42], with addition of purified siderophores to the growth media alleviating tungstate toxicity. Our transcriptomic data points to an increase in iron related gene expression in the presence of tungstate (figure 7B), particularly in the *baeR* mutant when tungstate is likely to be accumulating, and indicates a role for siderophores in the detoxification of tungstate in enteric organisms. In support of this hypothesis *S*. Typhimurium mutated in any of the *ent* genes, required for enterobactin synthesis and export (review see[43]), results in tungstate sensitivity on 30mM tungstate (Figure 8B). Furthermore, a *baeRfepA*double mutant, perturbed in the uptake of enterobactin, cannot be rescued by the proximity of the WT on tungstate plates (figure 8A). This process is branched to that of BaeR as *ent* genes provided *in trans* cannot complement the *baeR* mutant phenotype (data not shown).

## Discussion

The role of BaeR in the *Salmonella* response to envelope stress has not attracted as much attention, at least in the literature, as the major envelope stress counterparts RpoE and CpxR. This is possibly due, at first glance, to the lack of phenotypes that are obtained with the loss of BaeR under a wide range of conditions, particularly those that are associated with causing envelope damage. The lack of phenotypes associated with loss of BaeR is surprising given its classification as an envelope stress response pathway, and also given that the regulon members, away from the periplasmic chaperone, Spy, primarily consists of the RND multidrug efflux pumps MdtA andAcrD, which allow bacteria to resist a wide range of antimicrobial agents. What is/are the physiological inducing cues of *baeR*? In unpicking the answer to this question we sought to identify conditions which both induced the BaeR response and led to a *SalmonellabaeR* mutant phenotype.

BaeR has been implicated with the bacterial response to indole, with additions of 1mM indole leading to reduced cell viability in the absence of *baeR* in *E. coli* [8]. At cellular concentrations (µM) indole acts as a bacterial cell signalling molecule whilst at mM concentrations it is a biological oxidant. As we had observed no difference sensitivity of the *S*. Typhimurium*baeR* mutant to hydrogen peroxide we addressed the role of BaeR in the *Salmonella* response to indole. Whilst BaeRis moderately activated by mM concentrations of indole, we observed no difference between the survival rate of the *baeR* mutant and the isogenic parent strain. *E. coli* is an indole producer whilst *Salmonella* is not. We propose that the differences between the sensitivities of the *baeR* mutants in *Salmonella* and *E. coli* might lie between the differences in the indole producing/sensing pathways.

Previous studies also informed us that the BaeR response facilitates copper and zinc resistance [29], [34]. We screened a range of metals to look for inducers of the BaeR pathway, and agree that *baeR* in *S*. Typhimurium responds to increased exogenous copper and zinc concentrations. However, we found that out of the compared to zinc and copper, iron was a stronger inducer of *baeR* transcription. The paradox to this however, is that loss of BaeR does not correspond with toxicity to copper, zinc or iron indicating an ancillary role for fine tuning of the extremely complex and specific responses of bacteria to these metals.

Another novel inducer of BaeR is the clear induction at both the transcript and protein level upon entry into and survival in stationary phase growth, although loss of BaeR does not appear to contribute to premature death of stationary phase cultures. This is not the first envelope stress response pathway to be implicated in stationary phase survival. RpoE is maximally expressed upon entry into stationary phase [35], and PspA can compensate for loss of RpoE during stationary phase survival [10]. RpoE is also required for *Salmonella* to survive long term carbon starvation, where as CpxR is not [36]. It will be interesting to test whether BaeR is required for long term carbon starvation and whether it is also required for carbon starvation inducible cross resistance to other environmental stressed akin to RpoE.

We wanted to identify conditions in *S*. Typhimurium which induce the BaeR response, with loss of BaeR resulting in sensitivity to this condition, as route to try and identify the physiological role of this two component regulator. A previous study employing phenotype microarrays to unpick the functions of all two component systems in *E. coli*, identified myrcetin, gallic acid, nickel chloride and sodium tungstate, as growth inhibitors of a *baeR* mutant [38]. We repeated the phenotype analysis of the *S*. Typhimurium*baeR* mutant and identified sodium tungstate as the only condition out of almost 2000 where the *baeR* mutant did not survive as well as the isogenic parent. Validation of phenotype arrays analysis by other methods is extremely important to remove any bias from the culture conditions used in these microplate based assays. Confirmation of the *baeR* tungstate sensitivity and measurement of the largest transcriptional induction of *baeR* in the presence of sub-inhibitory concentrations of tungstate provided with us a tool to unpick the molecular regulation of the BaeR response.

The oxoanions molybdate and tungstate are chemically and physically very similar. However, the *baeR*mutant is not sensitive to high levels of molybdate and molybdate in molar excess cannot recover the tungstate phenotype (data not shown). Tungstate is best characterised as a selective inhibitor of a range of molybodenzymes including nitrate reductases, formatate dehyrogenases and xanthine oxidases, in each case tungstate acting as an agonist of molybdate (for review see [44]). However, some bacterial species have specific tungstate transporters such as TupA or WtpA, and tungstate can be selectively incorporated into some tungstoenzymes [44]. *Campyobacter jejuni*, for example, encodes an ultra high affinity tungstate transporter which facilitates tungsten incorporation into formate dehydrogenase [45]. *E. coli* and relatives have no such specific transporters but can transport tungstate into the cell through the molybdate ABC type transporters, ModA [46]. *Salmonella* has no known tungstoenzymes, although can survive in environments such as soil and water which can fluctuate in molybdate and tungstate concentrations. Under these conditions tungstate may then accumulate in the cell and require removal if levels become toxic. Indeed even in the chicken caeca, where *Salmonella* can colonise, tungstate is present in the nM range [45].

Why is *baeR* sensitive to tungstate? The tungstate sensitivity experiments were performed with aerobic, rich cultures of *Salmonella* where none of the molybdoenzymes are known to be essential for growth, so it is unlikely that this is the cause for cell death. We screened a number of strains with mutations in molybdoenzymes, for example nitrate reductase which is the classic example of a molydoenzyme disrupted by tungstate [47]. A mutant of *narG* and a triple mutant of all nitrate reductases grew like the wild type strain in the presence of tungstate, and there was no difference in nitrate reductase activity determined by methyl viologen assays between WT *Salmonella* and the *baeR* mutant (data not shown). A *S*. Typhimurium*rpoE* mutant has reduced cell viability when grown on sodium tungstate (Figure 6) and supports the notion that sodium tungstate, as well as disrupting enzyme function, also leads to envelope damage. We predicted that as BaeR had already been shown to regulate RND efflux pumps, although single mutants were themselves tungstate resistant, they must play an important role in the cellular response to tungstate. Microarray analysis of the WT and *baeR* mutant in the presence and absence of tungstate supported this prediction, with several efflux pumps up-regulated upon tungstate addition with a clear BaeR dependency, whilst genomic screening identified the *mdtABCDbaeSR* operon and *acrD* as the only loci which could complement the *baeR* tungstate phenotype. These observations, combined with loss of TolC also causing a tungstate sensitive phenotype, led us to construct multiple mutations in *mdtA*-*acrB* and *mdtA*-*acrD*, which do result in tungstate sensitivity. It is worth noting that CpxR also controls regulation of MdtA and AcrD, yet a CpxR mutant is not tungstate sensitive, indicating that BaeR is the primary regulator, at least in the terms of tungstate waste disposal. A *cpxR-baeR* double mutant is sensitive to tungstate at lower mM tungsate concentrations than a single *baeR*mutant (data not shown), suggesting some contribution to tungstate detoxification but only as an ancillary role to BaeR. Whether CpxR, or indeed many of the other regulators of drug efflux such as RamA or RamR, can compensate for BaeR under other stress inducing conditions remains to be elucidated, but the lack of BaeR phenotypes under these conditions indicates that this might be the case. For example AcrA and AcrB are required for *Salmonella* infection of macrophages and epithelia cells [48], whilst a BaeR mutant is not, indicating control by other regulatory mechanism in these environments. Control of drug efflux is increasingly complex and how BaeR fits into this regulatory network warrants further investigation.

In searching for the answer to explain why a *baeR* mutant is sensitive to tungstate we uncovered an alternative route for tungstate detoxification in *Salmonella*. Enterobactin siderophores are critically required for *Salmonella* to resist tungstate toxicity and mimics a model described for catecholate siderophores in protection of *Azotobacter vinelandii* from tungstate [41], [42]. The evidence that *Salmonella* has evolved and retained more than one mechanism for tungstate protection and/or detoxification indicates that *Salmonella* must indeed encounter this oxoanion during its lifecycle as discussed above.

Aside from the novel induction of BaeR by tungstate, stationary phase growth and iron we have also shown that BaeR is not required to establish murine Typhoid in either ity^S^ or ity^R^ mice. This is the first incidence where an envelope stress regulator is not required under these infection conditions. BaeR has been implicated with the colonisation of calves by *Salmonella* Dublin [32], and is likely to reflect differences in the local environment in the calf versus the mouse rather than specific infection mechanisms.

Whilst this manuscript was in preparationand in agreement with our study, LeBlanc and colleagues [49] demonstrate that *baeR* is induced by sodium tungstate in *E. coli*. However, the authors suggest that the physiological role for BaeR is to respond to stress which specifically damage MdtA leading to an induction of MdtA transport and removal of the toxic agent from the cell. Nevertheless, this study based on transcriptional fusions does not account for the lack of phenotypes associated with loss of BaeR, and our observations show that single mutants of the RND efflux pumps are not tungstate sensitive. Only when more than one of the RND systems is deleted, tungstate sensitivity is achieved. This leads us to suggest a modified role for BaeR, in the up-regulation of several RND systems including MdtAandAcrD in response to specific envelope damaging agents such as tungstate, as a belt and braces approach to protect the cell through waste disposal. In support of this, loss of BaeR in the presence of sub-inhibitory concentrations of tungstate results in up-regulation of the majority of envelope stress pathways. Linked with this observation, BaeR induction during stationary phase growth may well be to prevent accumulation of membrane damaging toxic metabolites, but requires further investigation.

## Materials and Methods

### Ethical Statement

The animal work in this study, project licence number PPL 80/1964, was approved by the University of East Anglia Ethical Review Committee (comprising lay members and scientific experts) and the Home Office Science and Research Group. All work was carried out in strict accordance with the regulations of Animals (Scientific Procedures) Act 1986 in Great Britain. All efforts were made to reduce group size, but still retain statistical significance, including use of a competition assay. All efforts were made to minimise suffering.

### Bacterial strains and media

Bacterial strains and plasmids used in this study are described in Table S1.Minimal medium 5.8 [50], Luria-Bertani (LB), or LB agar plates were complemented with the appropriate antibiotic at the concentrations of 50ug/ml kanamycin, 20ug/ml chloramphenicol, 100ug/ml ampicilin and 5ug/ml tetracycline. For the transcription profiling, the cells were grown in LB media to an OD_600_ = 1 supplemented with or without 20mM sodium tungstate.

To assay β-galactosidase, cells pre-grown over-night in LB were diluted 10^−2^ in LB media with or without: 20mM sodium tungstate; 2mM Indole; 1mM ferrous citrate; 0.5mM gallic acid; 1mM copper sulphate; 0.5mM zinc sulphate, and incubated to OD_600_ = 1, prior to being assayed as in [51].

### RNA and DNA extraction and quantification

At the appropriate OD_600_ and prior centrifugation, the cells were left on ice for a minimum of 30 min in a 1% (v/v) phenol (pH 4.3), 20% (v/v) ethanol solution to stabilise the mRNA[52]. Total RNAs were isolated using a SV Total RNA Isolation kit (Promega). Chromosomal DNA was isolated from strain, *S*. Typhimurium SL1344, using a Qiagen Genomic DNA isolation kit according to the manufacturer’s instructions. DNA and RNA samples were quantified at A_260_ and A_280_ using a NanoDrop 2000c (Thermo Scientific). The quality of RNA samples were assessed by size chromotography on an Experion RNA StdSens Chip (Bio-RAD) using the Experion Automated Electrophoresis Station (Bio-RAD) according to the manufacturer’s instructions.

### Microarrays labelling and hybridisation

Total RNA (10 µg) from at least 3 biological replicates was converted to cDNA and fluorescently labelled by random priming to incorporate Cy5-dCTP (Amersham) using reverse transcriptase (StrataScript, Stratagene). The labelled cDNA was mixed with 1/5 of 2 µg of chromosomal DNA labelled with Cy3 dCTP using the Gibco Bioprime DNA labelling System and hybridised on Agilent custom made 17K oligonucleotides arrays. Hybridisations were carried in a 50 µl volume per array. 21.5 µL of denatured CyDye label cDNA was mixed with 37.5 µL of hybridisation buffer (Morpholine-4-ethanesulfonic acid (MES) hydrate (pH 6.5) (Sigma, M2933) 50 mM, Sodium chloride 1 M, 99% Formamide (Sigma, F5786) 20% (w/v), EDTA 20 mM, Triton X-100 (Sigma, T8532) 1% (w/v)) and loaded onto the GASKET slide. An OGT array slide was then placed in contact with the hybridisation mix. In a tight chamber, the arrays were hybridised at 55°C for 60 hours in a light tight hybridisation oven at 8 rpm. The slide was removed from the hybridisation chamber and washed with buffer 1 (6XSSPE, 0.005% N-lauryl sarcosine) for 5min under agitation and for 5 min with the wash buffer 2 (0.06X SSPE, 0.18% polyethylene glycol 200) then dried in a centrifuge.

### Microarray data analysis

Slides were scanned using a GenePix 4000A scanner (Axon Instruments, Inc.). Fluorescent spots and local background intensities were quantified using Genepix Pro 7.0 Software (Axon Instruments, Inc.). The data were filtered so that spots with a reference signal lower than the background plus two standard deviations of the background or obvious blemishes were not included in the analysis. Signal intensities were corrected by subtracting the background and the red/green (Cy5/Cy3) ratios were calculated. Data were normalised using the Batch Anti-Banana Algorithm in R (BABAR) algorithm and software package which uses cyclic loess to normalise across the complete dataset[53]. Data from each microarray that passed the quality control procedures were then analyzed using Gene Spring 7.3 (Agilent) and statistically analysed using rank product analysis[54]. The expression data have been deposited in the NCBI Gene Expression Omnibus http://www.ncbi.nlm.nih.gov/geo/query/acc.cgi?acc=GSE28861 website and are accessible through GEO Series accession number GSE28861. All microarray data is MIAME compliant.

### Real time RT-PCR

The total RNAs were first treated with Turbo DNAseFree^TM^ from Ambion and the absence of DNA contamination was verified by PCR. 2ug of DNAseI-treated total RNA were retro-transcribed from random hexamers (Invitrogen) with the Superscript II^TM^ RT (Invitrogen) according to the manufacturer’s recommendations. Specific primers for the genes of interest amplifying an average product of 100 bp with about 60°C Tm were designed. The real-time PCR quantifications were realized on a 5 times dilution of the total cDNA obtained, using the Bio-Rad CFX96™ instrument and SensiMix^TM^ SYBR No-ROX kit (Bioline). The real-time PCR experiments were performed in triplicates, with 3 independent total RNA preparations. The calculated threshold cycle (Ct) for each gene was normalized to Ct of the *ampD* gene,which expression is invariant across large range of growth conditions.

### Western blot analysis

Equal amounts of protein from *S*. Typhimurium cell extracts were separated by 15% SDS-PAGE using a Mini Protean 3 electrophoresis system (Biorad) according to the manufacturer instructions. For immunoblotting, samples were transferred to nitrocellulose Biodyne A membranes (Pall Corporation) using a Mini Trans-Blot Electrophoretic cell (BioRad) according to the manufactures instructions. The proteins were fixed with methanol and the membranes were blocked with 5% skim milk and 1% BSA. Immobilized protein was detected using monoclonal 6xHis HRP conjugate antibody (1∶5000). 6xHis-taggedBaeR was detected using a luminol-based chemiluminescent detection system (Qiagen).

### Construction of a genomic library of *E. coli* BL21

To make the genomic library of *E. coli* BL21, 2.5 µg of genomic DNA was partially digested with *Eco*RI and then ligated to 1 µg of *Eco*RI-digested, dephosphorylated pLAFR3 cosmid DNA [55]. Of the resulting ligated DNA, 0.7 µg was packaged into recombinant λ phage using Gigapack III XL packaging extract (*Stratagene*) according to manufacturers’ specifications. The packaged DNA was transfected into *E. coli* strain 803, selecting for pLAFR3-containing transformants on LB tetracycline plates. Titering of the packaging mix was used to determine the number of primary transfectants (∼20,000). After amplification, *E. coli* primary transfectants were pooled into a single culture and stored in 25% glycerol at −80°C.

Cosmids were conjugated from *E. coli* 803 into *S.* Typhimurium SL1344 *baeR* by triparental mating using the helper plasmid pRK2013 to facilitate conjugal crosses[56].

### Plasmid construction

The *baeR*, *tolC* and *acrD*genes and the promoter region of the *mdtA* gene were PCR amplified from *S*. Typhimurium SL1344 genomic DNA using primers listed in supporting information Table S2. The PCR products were digested, ligated into the pBAD/Myc-His; the pBR322 and the pMP220 plasmids respectively, and transformed into *E. coli* strain TOP10 (Invitrogen™) by electroporation [57]. The resulting plasmids were designated and were confirmed by sequencing and are shown in supporting information Table S1.

### Phenotype Microarrays (Biolog)

Phenotypic characteristics were assayed using Biolog's phenotype microarray (PM) system (Hayward, CA), allowing high throughput screening of bacterial respiration response against a range of metabolic effectors including antimicrobials [37]. Phenotype analysis was performed using 20 PM panels containing various carbon sources (PM01-02), nitrogen sources (PM03), phosphorus and sulphur sources (PM04), biosynthetic pathway substrates (PM05), peptide nitrogen sources (PM06–08),osmolyte conditions (PM09), pH conditions (PM10), antimicrobials (PM11–14) and other metabolic effectors (PM15–20). All fluids, reagents and PM panels were used according to the manufacturer's instructions. Briefly, bacterial strains were cultured for 16 h on LB-G agar plates at 37^o^C, aerobically. Cells were harvested from the agar surface and adjusted to 85% transmittance (T) in inoculating fluid (IF-0a). Prior to addition to PM panels, bacterial suspensions were further diluted into 12 ml of IF-0a (PM01–08)or IF-10a PM09–20) inoculating fluid, supplemented with a final concentration of 0.02 M sodium succinate/0.002 mM ferric citrate (except PM01–02). Nutrient ource utilisation panels were supplemented with 0.0021% (w/v) histidine. Responses to each condition was measured via cellular respiration using a tetrazolium dye A, supplied by Biolog Inc (Hayward, USA). All microplates were incubated in the Omnilog instrument at 37^o^C and monitored for colour change at 15 min intervals for 48 hours. Kinetic data was analysed with OmniLog-PM software (Biolog). All experiments were performed once and comparisons between strains were based on the average of the area under the curve values at 48 hours.

### Construction of deletion mutants

For each deletion mutant, the entire structural genes were replaced by PCR-generated antibiotic resistance cassettes on the *S.* Typhimurium chromosome. Briefly, either a chloramphenicol or kanamycin resistance cassette was amplified by PCR from pKD4 and pKD3 plasmid templates using primers listed in Table S2. Each of these primers includes at its 5′ ends a 40 base-long extension showing homology with the flanking regions the target gene. The PCR products were used to replace the coding sequence of the target genes on the chromosome using the Lambda Red recombination system [58]; recombinant were selected for antibiotic resistance and verified by analytical PCR. All deletions were subsequently transduced with P22-phage into a clean background to avoid possible non-intended recombination events [59]. For some mutants, this antibiotic gene was removed using the pCP20 plasmid [58], [60].

### Construction of BaeR-6xHis

The C-terminal tagging of BaeR with the 6xHis epitope was performed using a modified λ Red method[61]. The DNA fragment to be recombined into the chromosome was PCR-amplified from the pSUB7 plasmid. The successful fusion of BaeR with the 6xHis epitope was confirmed by PCR and Western blot. All primers used to generate the different constructs are shown in Table S2.

### Mouse studies

For all *in vivo* studies, strains were grown statically overnight at 37°C, centrifuged, washed, resuspended to the appropriate concentration in sterile phosphate-buffered saline, and administered to mice in doses of 200 µl. Female BALB/c mice (6 to 8 weeks old; Charles River, UK) were used throughout. A competition index assay [62], where ∼10^3^ CFU each of the parent strain and isogenic *baeR*mutant were administered in a 1∶1 ratio via the intra-peritoneal route and CFU/organ determined at 72 hours post infection. For oral infection, single inoculums (∼5×10^5^) were administered via oral gavage, and mice were culled 5 days later. Organs (livers, spleens, and mesenteric lymph nodes) were isolated and homogenized, and numbers of bacteria present were enumerated by viable counting.

## Supporting Information

Table S1 List of strains and plasmids used in this study.
(DOCX)

TableS2 List of oligonucleotides used in this study.
(DOCX)

DataS1 Normalised log of ratio of the microarray results in LB and Tungstate 20mM for *Salmonella* SL1344 and *baeR* mutant.
(XLSX)
