# Supplementary material for: Novel Inducers of the Envelope Stress Response BaeSR in Salmonella Typhimurium: BaeR Is Critically Required for Tungstate Waste Disposal
Source: PLoS One. 2011 Aug 23;6(8):e23713. doi: 10.1371/journal.pone.0023713 (PMC3160322; doi:10.1371/journal.pone.0023713)
Supplement: Table S1 — List of strains and plasmids used in this study. (DOCX) [file pone.0023713.s001.docx]

Table S1. Bacterial strains and plasmids

| Strain/plasmid | Description | Reference |
| --- | --- | --- |
| *E. coli* strain 803 | *E. coli* host used for transformation of large plasmids, Met^-^ | [1] |
| *E. coli* strain BL21 | Derivative of *E. coli* strain B, conventionally used as a host for pET vectors | [2] |
| TOP10 | *E. coli* TOP10 | Invitrogen™ |
| MG1655 | *E. coli* K12 MG1655 | [3] |
| PT4 | *S. enterica* serovar Enteritidis str. P125109 | [4] |
| NCTC 12023 | Wild type (NCTC deposition of ATCC 14028) | D. Holden lab |
| SL1344 | *S. enterica* serovar Typhimurium 4/74 *hisG rpsL* | [5] |
| GVB311 | SL1344 ΔrpoE::kan | [6] |
| GVB368 | SL1344 *ΔcpxR::kan* | [7] |
| SMS438 | SL1344 *ΔrpoS::kan* | [8] |
| GR0001 | MG1655 *ΔbaeSR::kan* | This work |
| GR0002 | PT4 *ΔbaeSR::kan* | This work |
| GR0003 | NCTC 12023 *ΔbaeSR::kan* | This work |
| GR0004 | SL1344 Δ*baeS* | This work |
| GR0005 | SL1344 Δ*baeR* | This work |
| GR0006 | SL1344 Δ*baeR* pBaeR (Ap^R^) | This work |
| GR0007 | SL1344 *ΔbaeSR* | This work |
| GR0008 | SL1344 *ΔmdtABCE::cat* | This work |
| GR0009 | SL1344 *ΔSTM2133 - STM2135::kan* | This work |
| GR0010 | SL1344 *ΔSL0547 to SL0549* | This work |
| GR0011 | SL1344 *ΔcpxARΔ baeR* | This work |
| GR0012 | SL1344 *Δspy::kan* | This work |
| GR0013 | SL1344 *ΔcpxPΔ spy::kan* | This work |
| GR0014 | SL1344 *ΔcpxP::cat* | This work |
| GR0015 | SL1344 *Δspy::kan ΔzraP::cat* | This work |
| GR0016 | SL1344 *ΔyjaH::kan* | This work |
| GR0017 | SL1344 *ΔzraP::cat* | This work |
| GR0018 | SL1344 *ΔzraSR::kan* | This work |
| GR0019 | SL1344 *ΔyjaH -zraR::kan* | This work |
| GR0020 | SL1344 *ΔcadAB::kan* | This work |
| GR0021 | SL1344 *ΔbaeR ΔzraSR::kan* | This work |
| GR0022 | SL1344 *ΔbaeR ΔzraP::cat* | This work |
| GR0023 | SL1344 Δ*baeR ΔyjaH - zraSR::kan* | This work |
| GR0024 | SL1344 *ΔnirB::kan* | This work |
| GR0025 | SL1344 *ΔbaeS ΔcpxAR::kan* | This work |
| GR0026 | SL1344 *ΔbaeSΔ zraSR::kan* | This work |
| GR0027 | SL1344 *ΔtolC::kan* | This work |
| GR0028 | SL1344 *ΔacrD::kan* | This work |
| GR0029 | SL1344 *Δhnr::kan* | This work |
| GR0030 | SL1344 *ΔompW::kan* | This work |
| GR0031 | SL1344 *ΔacrAB::kan* | This work |
| GR0032 | SL1344 *ΔSL3010::kan* | This work |
| GR0033 | SL1344 *ΔyicO::kan* | This work |
| GR0034 | SL1344 *ΔSL4195::kan* | This work |
| GR0035 | SL1344 *Δspy ΔcpxP::cat Δ zraP::kan* | This work |
| GR0036 | SL1344 Δ*napABCD ΔnarGHIJ::cat ΔnarWXYZ::kan* | This work |
| GR0037 | SL1344 *Δfnr::kan* | This work |
| GR0038 | SL1344 *ΔnarZWXY* | This work |
| GR0039 | SL1344 Δ*narJI* | This work |
| GR0040 | SL1344 Δ*napABCD* | This work |
| GR0041 | SL1344 Δ*baeR Δfnr::kan* | This work |
| GR0042 | SL1344 Δ*baeR ΔSL3010::kan* | This work |
| GR0043 | SL1344 Δ*baeR ΔompW::kan* | This work |
| GR0044 | SL1344 Δ*feoA::kan* | This work |
| GR0045 | SL1344 Δ*baeR ΔfeoA::kan* | This work |
| GR0046 | SL1344 Δ*tolC* p*TolC* (Ap^R^) | This work |
| GR0047 | SL1344 Δ*baeR* pTol*C* (Ap^R^) | This work |
| GR0048 | SL1344 *ΔbaeR ΔtolC::kan* | This work |
| GR0049 | SL1344 *ΔarcA::cat* | This work |
| GR0050 | SL1344 *ΔarcA::cat Δfnr::kan* | This work |
| GR0051 | SL1344 *Δcrp::kan* | This work |
| GR0052 | SL1344 *ΔdcuB::kan* | This work |
| GR0053 | SL1344 *ΔsodC1::kan* | This work |
| GR0054 | SL1344 *ΔsodC2::cat* | This work |
| GR0055 | SL1344 *Δfur::cat* | This work |
| GR0056 | SL1344 *ΔtatABC::kan* | This work |
| GR0057 | SL1344 *ΔnirC::kan* | This work |
| GR0058 | SL1344 *ΔfhuF::kan* | This work |
| GR0059 | SL1344 *ΔsoxRS::kan* | This work |
| GR0060 | SL1344 *ΔcirA::kan* | This work |
| GR0061 | SL1344 *ΔbaeR ΔfhuF::kan* | This work |
| GR0062 | SL1344 *ΔbaeR ΔsoxRS::kan* | This work |
| GR0063 | SL1344 *ΔbaeR ΔcirA::kan* | This work |
| GR0064 | SL1344 *ΔentCEBA::kan* | This work |
| GR0065 | SL1344 *ΔiroBCDE::kan* | This work |
| GR0066 | SL1344 *ΔfepA::kan* | This work |
| GR0067 | SL1344 *ΔfepA::cat* | This work |
| GR0068 | SL1344 *ΔyncJ::kan* | This work |
| GR0069 | SL1344 *ΔiroN::kan* | This work |
| GR0070 | SL1344 *ΔiroN::cat* | This work |
| GR0071 | SL1344 *ΔtonB ::kan* | This work |
| GR0072 | SL1344 *ΔyedYZ::kan* | This work |
| GR0073 | SL1344 *ΔdppA::kan* | This work |
| GR0074 | SL1344 *ΔlamB malK::kan* | This work |
| GR0075 | SL1344 *ΔphsA::kan* | This work |
| GR0076 | SL1344 *ΔtdcABCDEG::kan* | This work |
| GR0077 | SL1344 *ΔSL1720::kan* | This work |
| GR0078 | SL1344 *ΔSL3786::kan* | This work |
| GR0079 | SL1344 *ΔosmB::kan* | This work |
| GR0080 | SL1344 *ΔwcaABCDEFGHI::kan* | This work |
| GR0081 | SL1344 *ΔgarDLRK::::kan* | This work |
| GR0082 | SL1344 *ΔbaeR ΔiroBCDE::kan* | This work |
| GR0083 | SL1344 *ΔbaeR ΔfepA::kan* | This work |
| GR0084 | SL1344 *ΔbaeR ΔyncJ::kan* | This work |
| GR0085 | SL1344 *ΔbaeR ΔiroN::kan* | This work |
| GR0086 | SL1344 *ΔbaeR ΔtonB::kan* | This work |
| GR0087 | SL1344 *ΔbaeR ΔyedYZ::kan* | This work |
| GR0088 | SL1344 *ΔbaeR ΔdppA::kan* | This work |
| GR0089 | SL1344 *ΔbaeR ΔlamB ΔmalK::kan* | This work |
| GR0090 | SL1344 *ΔbaeR ΔphsA::kan* | This work |
| GR0091 | SL1344 *ΔbaeR ΔtdcABCDEG::kan* | This work |
| GR0092 | SL1344 *ΔbaeRΔ SL1720::kan* | This work |
| GR0093 | SL1344 *ΔbaeR ΔSL3786::kan* | This work |
| GR0094 | SL1344 *ΔbaeR ΔosmB::kan* | This work |
| GR0095 | SL1344 *ΔbaeR ΔwcaABCDEFGHI::kan* | This work |
| GR0096 | SL1344 *ΔbaeR ΔgarDLRK::kan* | This work |
| GR0097 | SL1344 *ΔbaeR ΔentCEBA::kan* | This work |
| GR0098 | SL1344 *Δrob::kan* | This work |
| GR0099 | SL1344 *ΔentD::kan* | This work |
| GR0100 | SL1344 *ΔentF::kan* | This work |
| GR0101 | SL1344 *ΔfepB::kan* | This work |
| GR0102 | SL1344 *ΔcstA::kan* | This work |
| GR0103 | SL1344 *ΔoxyR::kan* | This work |
| GR0104 | SL1344 *ΔhtpX::kan* | This work |
| GR0105 | SL1344 *ΔacrEF::kan* | This work |
| GR0106 | SL1344 *ΔznuA::kan* | This work |
| GR0107 | SL1344 *ΔdmsA::kan* | This work |
| GR0108 | SL1344 *ΔpstCAB::kan* | This work |
| GR0109 | SL1344 *Δpsd::kan* | This work |
| GR0110 | SL1344 *ΔybjG::kan* | This work |
| GR0111 | SL1344 *ΔSL2756::kan* | This work |
| GR0112 | SL1344 *ΔychH::kan* | This work |
| GR0113 | SL1344 *ΔbaeR Δrob::kan* | This work |
| GR0114 | SL1344 *ΔbaeR ΔentD::kan* | This work |
| GR0115 | SL1344 *ΔbaeR ΔentF::kan* | This work |
| GR0116 | SL1344 *ΔbaeR ΔfepB::kan* | This work |
| GR0117 | SL1344 *ΔbaeR ΔcstA::kan* | This work |
| GR0118 | SL1344 *ΔbaeR ΔoxyR::kan* | This work |
| GR0119 | SL1344 *ΔbaeR ΔhtpX::kan* | This work |
| GR0120 | SL1344 *ΔbaeR ΔacrEF::kan* | This work |
| GR0121 | SL1344 *ΔbaeR ΔznuA::kan* | This work |
| GR0122 | SL1344 *ΔbaeR ΔdmsA::kan* | This work |
| GR0123 | SL1344 *ΔbaeR ΔpstCAB::kan* | This work |
| GR0124 | SL1344 *ΔbaeR Δpsd::kan* | This work |
| GR0125 | SL1344 *ΔbaeR ΔybjG::kan* | This work |
| GR0126 | SL1344 *ΔbaeR ΔSL2756::kan* | This work |
| GR0127 | SL1344 *ΔbaeR ΔychH::kan* | This work |
| GR0128 | SL1344 *ΔSTM2804::kan* | This work |
| GR0129 | SL1344 *ΔyebB::kan* | This work |
| GR0130 | SL1344 *ΔnixA::kan* | This work |
| GR0131 | SL1344 *ΔyeeN::kan* | This work |
| GR0132 | SL1344 *ΔemrAB ::kan* | This work |
| GR0133 | SL1344 *ΔmacAB::kan* | This work |
| GR0134 | SL1344 *ΔyjbB::kan* | This work |
| GR0135 | SL1344 *ΔygaE::kan* | This work |
| GR0136 | SL1344 *ΔcyoA::kan* | This work |
| GR0137 | SL1344 *ΔmdfA::kan* | This work |
| GR0138 | SL1344 *ΔiroN::cat ΔcirA::kan* | This work |
| GR0139 | SL1344 *ΔiroN::cat ΔfepA::kan* | This work |
| GR0140 | SL1344 *ΔcirA::kan ΔfepA::cat* | This work |
| GR0141 | SL1344 *ΔmdtABCE::cat ΔacrAB::kan* | This work |
| GR0142 | SL1344 *ΔmdtABCE::cat ΔacrD::kan* | This work |
| GR0143 | SL1344  *ΔbaeR* pacrD (Ap^R^) | This work |
| GR0146 | SL1344  *ΔbaeR Δfur::cat* | This work |
| GR0147 | SL1344 *ΔacrAB::catΔacrD::kan* | This work |
| Plasmids: |  |  |
| pLAFR3 | Wide host-range cosmid cloning vector (Tc^R^) | [9] |
| pRK2013 | Used as mobilising plasmid in tri-parental crosses (Kan^R^) | [10] |
| pSUB7 | For construction of C-terminal 6xHis tags on the chromosome, pGP704 derivative (Kan^R^) | [11] |
| pKD3 | pANTSγ derivative (Cm^r^) | [12] |
| pKD4 | pANTSγ derivative (Km^r^) | [12] |
| pKD46 | pBAD18 derivative (Amp^r^) | [12] |
| pCP20 | temperature-sensitive replication and thermal induction of FLP synthesis (Ap^R^, Cm^r^ ) | [13] |
| pBAD/*Myc*-His A | C-Terminal 6xHis Tags, (Ap^R^) | Invitrogen™ |
| pMP220 | Wide host-range promoterless-*lacZ* probe vector (Tet^R^) | [14] |
| pBR322 | Low copy cloning vector (Ap^R^, Tc^R^) | [15] |
| pBaeR | *baeR* gene in pBaD/Myc-His expression plasmid without the 6xHis tag (Ap^R^) | This work |
| pTolC | *tolC* gene in pBaD/Myc-His expression plasmid without the 6xHis tag (Ap^R^) | This work |
| pPmdtA | promoter of *mdtA* fused with promoterless-*lacZ* in pMP220 (Tc^R^) | This work |
| pacrD | *acrD* structural gene in pBR322 (Ap^R^) | This work |

1. Wood WB (1966) Host specificity of DNA produced by Escherichia coli: bacterial mutations affecting the restriction and modification of DNA. J Mol Biol 16: 118-133.

2. Studier FW, Moffatt BA (1986) Use of bacteriophage T7 RNA polymerase to direct selective high-level expression of cloned genes. J Mol Biol 189: 113-130.

3. Blattner FR, Plunkett G, 3rd, Bloch CA, Perna NT, Burland V, et al. (1997) The complete genome sequence of Escherichia coli K-12. Science 277: 1453-1462.

4. Thomson NR, Clayton DJ, Windhorst D, Vernikos G, Davidson S, et al. (2008) Comparative genome analysis of Salmonella Enteritidis PT4 and Salmonella Gallinarum 287/91 provides insights into evolutionary and host adaptation pathways. Genome Res 18: 1624-1637.

5. Hoiseth SK, Stocker BA (1981) Aromatic-dependent Salmonella typhimurium are non-virulent and effective as live vaccines. Nature 291: 238-239.

6. Humphreys S, Stevenson A, Bacon A, Weinhardt AB, Roberts M (1999) The alternative sigma factor, sigmaE, is critically important for the virulence of Salmonella typhimurium. Infect Immun 67: 1560-1568.

7. Humphreys S, Rowley G, Stevenson A, Anjum MF, Woodward MJ, et al. (2004) Role of the two-component regulator CpxAR in the virulence of Salmonella enterica serotype Typhimurium. Infect Immun 72: 4654-4661.

8. O'Neal CR, Gabriel WM, Turk AK, Libby SJ, Fang FC, et al. (1994) RpoS is necessary for both the positive and negative regulation of starvation survival genes during phosphate, carbon, and nitrogen starvation in Salmonella typhimurium. J Bacteriol 176: 4610-4616.

9. Staskawicz B, Dahlbeck D, Keen N, Napoli C (1987) Molecular characterization of cloned avirulence genes from race 0 and race 1 of Pseudomonas syringae pv. glycinea. J Bacteriol 169: 5789-5794.

10. Figurski DH, Helinski DR (1979) Replication of an origin-containing derivative of plasmid RK2 dependent on a plasmid function provided in trans. Proc Natl Acad Sci U S A 76: 1648-1652.

11. Uzzau S, Figueroa-Bossi N, Rubino S, Bossi L (2001) Epitope tagging of chromosomal genes in Salmonella. Proc Natl Acad Sci U S A 98: 15264-15269.

12. Datsenko KA, Wanner BL (2000) One-step inactivation of chromosomal genes in Escherichia coli K-12 using PCR products. Proc Natl Acad Sci U S A 97: 6640-6645.

13. Cherepanov PP, Wackernagel W (1995) Gene disruption in Escherichia coli: TcR and KmR cassettes with the option of Flp-catalyzed excision of the antibiotic-resistance determinant. Gene 158: 9-14.

14. Zaat SA, Wijffelman CA, Spaink HP, van Brussel AA, Okker RJ, et al. (1987) Induction of the nodA promoter of Rhizobium leguminosarum Sym plasmid pRL1JI by plant flavanones and flavones. J Bacteriol 169: 198-204.

15. Bolivar F, Rodriguez RL, Greene PJ, Betlach MC, Heyneker HL, et al. (1977) Construction and characterization of new cloning vehicles. II. A multipurpose cloning system. Gene 2: 95-113.
