# Supplementary material for: Novel Inducers of the Envelope Stress Response BaeSR in Salmonella Typhimurium: BaeR Is Critically Required for Tungstate Waste Disposal
Source: PLoS One. 2011 Aug 23;6(8):e23713. doi: 10.1371/journal.pone.0023713 (PMC3160322; doi:10.1371/journal.pone.0023713)
Supplement: TableS2 — List of oligonucleotides used in this study. (DOCX) [file pone.0023713.s002.docx]

Supplementary Table S2. Primers used in this study

| Name | DNA sequence |
| --- | --- |
|  | **construction of deletion mutants** |
| baeSf | TTCGCCACCTGCATCGTGCTGCTGATCAGTATGCATTGGGCCGTGTAGGCTGGAGCTGCTTC |
| baeSr | ATGCTAACCCCGCCAAAAGGCGAATGAGCGGCGTGAAGATCATATGAATATCCTCCTTAG |
| baeRf | ATGACTGAATTACCCATTGATGAAAACACGCCGCGCATTTGTGTAGGCTGGAGCTGCTTC |
| baeRr | CTTCCCAGCGATATCCCACCCCGTAGACCGCGCGTATAAACATATGAATATCCTCCTTAG |
| spyF | atgcgtaaactgactgctctatttgttgcctctaccctggGTGTAGGCTGGAGCTGCTTC |
| spyR | ttctgccgcagcaggcattttaccttcttgcgccgggcgtCATATGAATATCCTCCTTAG |
| CpxP F | ATGCGCAAAGTTACCGCTGCTGTTATGGCCTCAACGCTGGGTGTAGGCTGGAGCTGCTTC |
| CpxP R | TTACTGGGAACGTGAGTTGCTACTACTCAATAATTTCAACCATATGAATATCCTCCTTAG |
| TatAF | ATGGGTGGTATCAGTATTTGGCAGTTGTTGATTGTTGCCGGTGTAGGCTGGAGCTGCTTC |
| TatCR | CCTTTTCGGTTTCGGCCTCGTTATCTTCGTCGCGCGTCCGCATATGAATATCCTCCTTAG |
| STM2126 F | atgaaaggcagtaatactttccgctgggcaatagcgattgGTGTAGGCTGGAGCTGCTTC |
| yegB R | CCGGGATATTGATCAGAAAGATCCAGTGCCATGAAGCATACATATGAATATCCTCCTTAG |
| STM2133_F | atggatttttatgacagaaacgtcatctcaccgctataaaGTGTAGGCTGGAGCTGCTTC |
| STM2133_R | ttacaccattgagttgacctgtcccggcagctctgaattcCATATGAATATCCTCCTTAG |
| ColibaeS_F | ACTGTTTCTGGCGATTTTCGCCACCTGCATTGTCTTGCTGGTGTAGGCTGGAGCTGCTTC |
| ColibaeR_F | ACACACCGCGTATTTTGATCGTGGAAGATGAACCGAAGCTGTGTAGGCTGGAGCTGCTTC |
| ColibaeR_R | AACGATGCGGCAGGCGTCGGCTTCCCAGCGGTAACCGACGCATATGAATATCCTCCTTAG |
| yjaH_R | gcttttcgcttgcggctgccagtgacaaaaatctgaattcCATATGAATATCCTCCTTAG |
| yjaH_F | cgcgctgttgctgactgcctgcagtcataacgcttcaccgGTGTAGGCTGGAGCTGCTTC |
| zraP_F | gaacaataaatcagctatcgcgctaattgccctctctcttGTGTAGGCTGGAGCTGCTTC |
| zraP_R | agtttcccatacccatgtgaccgccgccgcgatgataaccCATATGAATATCCTCCTTAG |
| zraSR_F | ggggctattttccattatggtcattcgcgactatggccgtGTGTAGGCTGGAGCTGCTTC |
| zraSR_R | gcagcgttttgcgcgtaatgcccaattgacgggcggcttcCATATGAATATCCTCCTTAG |
| cadBAL_F | gctatttgcctgtactggcgtcgttgccggtaatatgatgGTGTAGGCTGGAGCTGCTTC |
| cadBAL_R | gatgtagtccgggtgcaaacccatctcaggccgacaatggCATATGAATATCCTCCTTAG |
| hnr_F | ccattggtcggaaaacagattcttattgttgaagacgagcGTGTAGGCTGGAGCTGCTTC |
| hnr_R | gcagacaacatcaatcgcagacgccctcctgcgccccataCATATGAATATCCTCCTTAG |
| acrD_F | ttgcctgggtgctggctatcctgttgtgtctgacaggggcGTGTAGGCTGGAGCTGCTTC |
| acrD_R | ggcttcagcgggaagcggcgacgtatcagcacgaaaaacaCATATGAATATCCTCCTTAG |
| ompW_F | atttacagtggcggcactggcgttaacaactcttctctcaGTGTAGGCTGGAGCTGCTTC |
| ompW_R | gccgagaacataaatacccatgggtccagacgcacgctgtCATATGAATATCCTCCTTAG |
| SL3010_F | tagccggaagtaccggtattgttctgctgtctgctgcagcGTGTAGGCTGGAGCTGCTTC |
| SL3010_R | taacccacgccaagattaaaaccattgctattaaggcttcCATATGAATATCCTCCTTAG |
| yicO_F | ggggacgcaatcgttgccgtactaacaaattgagtgaaaaGTGTAGGCTGGAGCTGCTTC |
| yicO_R | gtcaataaacacaatcttcagcacaaataacagcgcgacaCATATGAATATCCTCCTTAG |
| acrAB_F | gttaacgcctctggcggtcgttctgatgctctcaggcagcGTGTAGGCTGGAGCTGCTTC |
| acrAB_R | tgtcgaatgactatgctcaatatcttcgcttttacggctaCATATGAATATCCTCCTTAG |
| SL4195_F | ctgcttttattacacaaagcacatacgccagcgagcttccGTGTAGGCTGGAGCTGCTTC |
| SL4195_R | atttccttttaaataatccattaaatgaaaaatcaatgagCATATGAATATCCTCCTTAG |
| sodC1_F | gctcatcagttgttcagcaatggcagagaataccctgactGTGTAGGCTGGAGCTGCTTC |
| sodC1_R | atgacaccacaggcaaaacgtgcaccgccaccacccagtgCATATGAATATCCTCCTTAG |
| sodC2_F | taagtttagcgatggtgacgctgctggcctgtgcgggtgcGTGTAGGCTGGAGCTGCTTC |
| sodC2_R | cggtttcggctgatcggacatgttatcgccgcccacatggCATATGAATATCCTCCTTAG |
| arcA_F | ttatcgttgaagacgagttggtaacacgcaacacgttgaaGTGTAGGCTGGAGCTGCTTC |
| arcA_R | gcgataaccttcgccgtgaatggtggcgatgatttccggcCATATGAATATCCTCCTTAG |
| fur_F | ataccgcattaaagaaggctggcctgaaagtaacgcttccGTGTAGGCTGGAGCTGCTTC |
| fur_R | cgtgcgcgtgctcgtcttcgcggcagtcgccttcagcgcaCATATGAATATCCTCCTTAG |
| dcuB_F | aatatgtctgttttatggtgcccgaaagggcgggatcgcgGTGTAGGCTGGAGCTGCTTC |
| dcuB_R | gacgctcacgccaatcaaacccggcagaatgaagctgtggCATATGAATATCCTCCTTAG |
| crp_F | acagacccgactcttgaatggttcttgtctcattgccacaGTGTAGGCTGGAGCTGCTTC |
| crp_R | tagacgacgatggtcttgccatgcgcggagatcaggttttCATATGAATATCCTCCTTAG |
| feoA_F | ctcctgacactgcgtggaaaatcaccggctttgcgcgtgaGTGTAGGCTGGAGCTGCTTC |
| feoA_R | tccacttctattaacgccaaatcctttttacgcaaaaccaCATATGAATATCCTCCTTAG |
| ent F | actggccgaggacgctcaggagacaatggcaacgcttgctGTGTAGGCTGGAGCTGCTTC |
| ent R | tatcctgcaaggtaatgtggctggccagatcggaggcaagCATATGAATATCCTCCTTAG |
| iro F | attctgtttgtcggtccaccactgtatggactgctataccGTGTAGGCTGGAGCTGCTTC |
| iro R | ctgcgaaaagaggcgttaaacatgggcccgtggcccaggcCATATGAATATCCTCCTTAG |
| fepA F | tcattccctgaccttactggtcaatttagggatttacgggGTGTAGGCTGGAGCTGCTTC |
| fepA R | atgctcatataccacgtacgtcctggttcgttataggtgtCATATGAATATCCTCCTTAG |
| yncJ F | atgcttacaaaaacgttatcagtagttttactgacctgtgGTGTAGGCTGGAGCTGCTTC |
| yncJ R | atagctttctggtttgcgcgatttctgccagttgtggtgcCATATGAATATCCTCCTTAG |
| iroN F | aaccgtggtttctacaggggttaatagtccattatcagcaGTGTAGGCTGGAGCTGCTTC |
| iroN R | ggttcgttataggtatttgccccttcggcggagcgatagaCATATGAATATCCTCCTTAG |
| tonB F | gtcgctttccctggccgacactcctttccgtaggcattcaGTGTAGGCTGGAGCTGCTTC |
| tonB R | tctgcgccgtaccgttcagacggaagataatattgaccacCATATGAATATCCTCCTTAG |
| yedYZ F | aagatacgtccattaacagaagccgatgtgactgcggaatGTGTAGGCTGGAGCTGCTTC |
| yedYZ R | ggcgaaacttccggtaacgcaacgctaaaagggctaacgcCATATGAATATCCTCCTTAG |
| dppA F | ttccttgaagaagtcagggatgctgaagcttggtttgagcGTGTAGGCTGGAGCTGCTTC |
| dppA R | gagacgttttcgaagtgatgtttgcctaatggatcaaccaCATATGAATATCCTCCTTAG |
| gar F | ctgcgctgcgcattcctatcgccagagcggcagcaacattGTGTAGGCTGGAGCTGCTTC |
| gar R | gtgcggggttaaacactgccagctggttatgcaatccccaCATATGAATATCCTCCTTAG |
| lam mal F | gtacagctacgaaatgtaacgaaagcctggggtgacgtggGTGTAGGCTGGAGCTGCTTC |
| lam mal R | atctgggcgccgaaggtccactcatcgctatcgccacggcCATATGAATATCCTCCTTAG |
| phsA F | ttgcagggggtaggcatcggctgctctgcctgcgcgctggGTGTAGGCTGGAGCTGCTTC |
| phsA R | cggctcagcgtcacgcctgcggtatgcactaccgtaccggCATATGAATATCCTCCTTAG |
| tdc F | tccctaaaacacagcatttggtggtctttcaggaagtcatGTGTAGGCTGGAGCTGCTTC |
| tdc R | acgactttaatcgccagaccgccgcgcgacgtctcgcgatCATATGAATATCCTCCTTAG |
| SL1720 F | ttgacttatcgcgctggcagtttgcgctgaccgcgctgtaGTGTAGGCTGGAGCTGCTTC |
| SL1720 R | cttgttgttgatgttgcatggcgctcggcccaagacgcgcCATATGAATATCCTCCTTAG |
| SL3786 F | ctctatgcgacaacagttatcaccgttgggctgttatgttGTGTAGGCTGGAGCTGCTTC |
| SL3786 R | tcaggttgatatggcgtataaacaccggtcaggctttccaCATATGAATATCCTCCTTAG |
| osmB F | gtttatgacgagcaaaaaaatggccgctgctgtgctggcaGTGTAGGCTGGAGCTGCTTC |
| osmB R | tttccaacctggtgaccaatcacgccgccgaccgccgcgcCATATGAATATCCTCCTTAG |
| wca F | cagacaatccgctgatctcaatttatatgccaacctggaaGTGTAGGCTGGAGCTGCTTC |
| wca R | aatatctgcaataaattggcgtagcacgttctctttattgCATATGAATATCCTCCTTAG |
| SL1720 F | ttgacttatcgcgctggcagtttgcgctgaccgcgctgtaGTGTAGGCTGGAGCTGCTTC |
| SL1720 R | cttgttgttgatgttgcatggcgctcggcccaagacgcgcCATATGAATATCCTCCTTAG |
| SL3786 F | ctctatgcgacaacagttatcaccgttgggctgttatgttGTGTAGGCTGGAGCTGCTTC |
| SL3786 R | tcaggttgatatggcgtataaacaccggtcaggctttccaCATATGAATATCCTCCTTAG |
| osmB F | gtttatgacgagcaaaaaaatggccgctgctgtgctggcaGTGTAGGCTGGAGCTGCTTC |
| osmB R | tttccaacctggtgaccaatcacgccgccgaccgccgcgcCATATGAATATCCTCCTTAG |
| wca F | cagacaatccgctgatctcaatttatatgccaacctggaaGTGTAGGCTGGAGCTGCTTC |
| wca R | aatatctgcaataaattggcgtagcacgttctctttattgCATATGAATATCCTCCTTAG |
| rob F | gcataattcgcgacctgttaatctggctggaaggtcatctGTGTAGGCTGGAGCTGCTTC |
| rob R | aatcgggatcagaaattcgcagcgcaggttgatagggcgaCATATGAATATCCTCCTTAG |
| entD F | cctttgccggacacaggctgcatatcgtcgactttgatgcGTGTAGGCTGGAGCTGCTTC |
| entD R | attgcgctaagtatagaagcgggagcgctcctgtcgtgggCATATGAATATCCTCCTTAG |
| entF F | gcgtttaccgttagtcgccgcccagccggggatctggatgGTGTAGGCTGGAGCTGCTTC |
| entF R | aagttcgctgatgatgggcccgatagtttcaaaagcggagCATATGAATATCCTCCTTAG |
| fepB F | ttatcgttggctgcttttagtggttggtatatcaatttcaGTGTAGGCTGGAGCTGCTTC |
| fepB R | ggcaagccggttaagcaacaatgttgcgctgtaataatccCATATGAATATCCTCCTTAG |
| cstA F | aatcagggaaatacctcgtctggacagcgctctcagtattGTGTAGGCTGGAGCTGCTTC |
| cstA R | ttcgcctgcgtcacgatctcatccacattttccggcatcgCATATGAATATCCTCCTTAG |
| oxyR F | tcgtgatcttgaatatctggtggcgttagccgaacatcgcGTGTAGGCTGGAGCTGCTTC |
| oxyR R | atggccatccattgcgccacggatggcctctgccagttgcCATATGAATATCCTCCTTAG |
| htpX F | atcgcgctcttcctgctgacgaacctggccgtgatggtcgGTGTAGGCTGGAGCTGCTTC |
| htpX R | gtactcgccgctacgcagcgcttcaatgcgtttatccagcCATATGAATATCCTCCTTAG |
| acrEF F | tgccaggttttcactcctgccctcattcatcatattctctGTGTAGGCTGGAGCTGCTTC |
| acrEF R | attaaatcggcgttttacaacaacgaagaataccggcacgCATATGAATATCCTCCTTAG |
| znuA F | cattacgatgattagtcgcattatgttacagaaaaatacgGTGTAGGCTGGAGCTGCTTC |
| znuA R | caggcagctcgcatactggttggctaattggcttaaaaacCATATGAATATCCTCCTTAG |
| dmsA F | atccctgatgccgtgctggcagccgaggtgagccgtcgtgGTGTAGGCTGGAGCTGCTTC |
| dmsA R | caacctgaacgaggttcgtatgtgacgggttccccttcgcCATATGAATATCCTCCTTAG |
| dnaG F | tggacgaatcccacgcgtattcattaatgacctgctggcgGTGTAGGCTGGAGCTGCTTC |
| dnaG R | aaccgtgagtgcgatcgcgagcgatcagctcttcttgtcgCATATGAATATCCTCCTTAG |
| pstCAB F | gcctgcttttaacccgccgggtaaaaaaggcgacatgattGTGTAGGCTGGAGCTGCTTC |
| pstCAB R | ttcgcgggcttggtgaacagatcgtccgtgttgctgaactCATATGAATATCCTCCTTAG |
| psd F | ttaaactttcgctacaatacattctgccgaaactatggctGTGTAGGCTGGAGCTGCTTC |
| psd R | cagcgggctggcgtcgtgttcggctttaatttcttctgccCATATGAATATCCTCCTTAG |
| ybjG F | caactcgctgttttattttattaacgccacgcctgattccGTGTAGGCTGGAGCTGCTTC |
| ybjG R | gtacccagccttttcgaatgggcaatgcgaaacagaaacgCATATGAATATCCTCCTTAG |
| SL2756 F | atcaacactaacagtctgtcgctgctgacccagaataaccGTGTAGGCTGGAGCTGCTTC |
| SL2756 R | agagacagcacgttctgcgggacctggttagcctgcgccaCATATGAATATCCTCCTTAG |
| ychH F | aacgcttcgttattcggtaacgtgctgatggggttggggcGTGTAGGCTGGAGCTGCTTC |
| ychH R | ctatgacgacgattgtcgttacggcggcaacgtttgtcatCATATGAATATCCTCCTTAG |
| STM2804 F | gtaaacctccgggctcagttcataatagggttggcgtaacGTGTAGGCTGGAGCTGCTTC |
| STM2804 R | cgatcgccagacggttaaagcagttcattaaaccgatggcCATATGAATATCCTCCTTAG |
| yebB F | aacattattctgtccaatatgaaaccggggatatcgtcttGTGTAGGCTGGAGCTGCTTC |
| yebB R | cctcgcgttgaggggtttcaattccacaggcgctaattaaCATATGAATATCCTCCTTAG |
| nixA F | actaaacgacggggaatagcattaaccgtctttcttgtcgGTGTAGGCTGGAGCTGCTTC |
| nixA R | tatcatagccgcgccagcgataattcaacaccgagagcagCATATGAATATCCTCCTTAG |
| yeeN F | tgggcaaatattgttgctaagaaaacagctaaagacggtgGTGTAGGCTGGAGCTGCTTC |
| yeeN R | gtttgcaacgttatgatatactttttgcacatcatcatcaCATATGAATATCCTCCTTAG |
| emrAB F | gcaaatgcggagatccaaaccccgcagcaaccggctaagaGTGTAGGCTGGAGCTGCTTC |
| emrAB R | gcaccgaagggcggtttggcgaaccacaccaggccgagcaCATATGAATATCCTCCTTAG |
| macAB F | agaaatttaaaaagcgttatctggtcattattttaattctGTGTAGGCTGGAGCTGCTTC |
| macAB R | cgcatccaccgggtccagtcgcgccgcgtttctcgccggtCATATGAATATCCTCCTTAG |
| yjbB F | tccatctactttctgccgttgcgctgttggtatggggaacGTGTAGGCTGGAGCTGCTTC |
| yjbB R | tgatccggctgttccagtacgctataggcgaccgaacagaCATATGAATATCCTCCTTAG |
| ygaE F | tcccaaccgacggccatagatggctatcgctggcttaaaaGTGTAGGCTGGAGCTGCTTC |
| ygaE R | aataatgggaatcggcgtcagcaaatgctgacgcattaatCATATGAATATCCTCCTTAG |
| cyoA F | actcaggaaatacaataaaagtttgggatggttgtcattaGTGTAGGCTGGAGCTGCTTC |
| cyoA R | attccgcgtggctcatgtccataccttccattccttcatgCATATGAATATCCTCCTTAG |
| mdfA F | atcaggcggcaggctgggacgtcaggcgctgctttttcctGTGTAGGCTGGAGCTGCTTC |
| mdfA R | cttatcttttaagaagaccagcatcagcagtagccaaagaCATATGAATATCCTCCTTAG |
|  | **RT Q-PCR** |
| RQ nirB F | CTACGGCTGCGAAGTGTGTA |
| RQ nirB R | AGTATCCTGTAGCGGCGTGT |
| RQnarG F | TGCTGGTGATGCTTGAAGAG |
| RQnarG R | CCATTCCGGGTTATTTTCCT |
| RQnapA F | GCGGACAACGGTATTGTCTT |
| RQnapA R | AATCCTGATTTACCGCGTTG |
| RQnapD F | TCAGAGTGAAACGCTGATGC |
| RQnapD R | ATGGTGTTTCCTCACCTTGC |
| RT ampD F | ATGACGAAAAACCGTCCTTG |
| RT ampD R | GGATCTATCGTTCCGGTGAA |
| RT spy F | GCCAATGATGCAGCATAAAG |
| RT spy R | TAATGTCGCGAATTTGTTGC |
| RT baeR F | CGAAAGCCCGTTAATGATTG |
| RT baeR R | TACCCGGTTCAAGCGATAAG |
| RT baeS F | GGGGCTAACGCTACATTACG |
| RT baeS R | AGCGCAGACTGTTTTCCAGT |
|  | **cloning in plasmids** |
| BaeR Nco F | taaCCATGGCTGAATTACCCATTGATGAA |
| BaeR Hind R | taaaagcttTTATACCAGGCGACACGCAT |
| acrD Hind F | aaaaAAGCTTaacgtaaagaacggcaaatc |
| acrD Sph R | aaaaGCATGCtgtgcccgacacctcgtatc |
| pnirB_Eco_F | aaaagaattcttgcgctcggctttgtatcc |
| pnirB_pst_R | aaaactgcagtcgatttcttttctattacc |
| pMdtA_Eco_F | aaaagaattctggcggcgatgatttcggctc |
| pMdtA_Pst_R | aaaactgcaggtattactgcctttcatcgg |
| TolC Xho F | aaaaaCTCGAGaatgaagaaattgctccccatc |
| TolC Eco R | aaaagaattcatcaatgccggaatggattg |
